# Chemokine Expression-Based Endotype Clustering of Chronic Rhinosinusitis

**DOI:** 10.3390/jpm12040646

**Published:** 2022-04-18

**Authors:** Ulrike Förster-Ruhrmann, Agnieszka J. Szczepek, Greta Pierchalla, Joachim W. Fluhr, Metin Artuc, Torsten Zuberbier, Claus Bachert, Heidi Olze

**Affiliations:** 1 Department of Otorhinolaryngology Head and Neck Surgery CVK, Charité—Universitätsmedizin Berlin, Corporate Member of Freie Universität Berlin and Humboldt-Universität zu Berlin, 13353 Berlin, Germany; ulrike.foerster@charite.de (U.F.-R.); greta.pierchalla@charite.de (G.P.); 2 Department of Otorhinolaryngology Head and Neck Surgery CCM, Charité—Universitätsmedizin Berlin, Corporate Member of Freie Universität Berlin and Humboldt-Universität zu Berlin, 10117 Berlin, Germany; 3 Faculty of Medicine and Health Sciences, University of Zielona Gora, 65-046 Zielona Gora, Poland; 4 Institute of Allergology, Charité—Universitätsmedizin Berlin, Corporate Member of Freie Universität Berlin and Humboldt-Universität zu Berlin, 10117 Berlin, Germany; joachim.fluhr@charite.de (J.W.F.); metartuc487@gmail.com (M.A.); torsten.zuberbier@charite.de (T.Z.); 5 Fraunhofer Institute for Translational Medicine and Pharmacology ITMP, Allergology and Immunology, 12203 Berlin, Germany; 6 Upper Airway Research Laboratory, Ghent University, 9000 Ghent, Belgium; claus.bachert@ugent.be

**Keywords:** chronic rhinosinusitis, nasal polyps, asthma, chemokines, cytokines, endotyping

## Abstract

Chronic rhinosinusitis (CRS) with (CRSwNP) or without nasal polyps (CRSsNP) is a persistent, heterogeneous inflammatory condition affecting the upper respiratory tract. The present study aimed to improve the characterization of CRS endotypes based on the chemokine and cytokine expression pattern in the CRS tissues. Concentrations of chemokines and cytokines were measured in tissues from nasal biopsies obtained from 66 CRS patients and 25 control subjects using multiplexing or single analyte technologies. Cluster analysis based on the concentration of type-1 (MCP-3/CCL7, MIP-1 α/CCL3), type-2 (IL-5, MCP-3/CCL7, MIP-1 α/CCL3, TARC/CCL17, PARC/CCL18, IP-10/CXCL10, ECP), and type-3 (IL-22) chemokines and cytokines identified six CRS endotypes (clusters). Cluster 1 (type-3) and 2 (type-1) were associated with a low prevalence of nasal polyps, Cluster 3 (type-1, -2, -3) and Cluster 4 (type-2, -3, medium IL-22) with medium, and Cluster 5 (type-2, -3, high Il-22) and Cluster 6 (type-2) with high prevalence of nasal polyps. Asthma was highly prevalent in Cluster-6. Our findings add to the existing knowledge of CRS endotypes and may be useful for the clinical decision-making process. The advancement of biologics therapy for upper respiratory tract disorders rationalizes the personalized diagnostic approach to warrant a successful treatment and monitoring of CRS.

## 1. Introduction

Chronic rhinosinusitis (CRS) impairs the quality of life and work productivity of the affected persons and causes an economic burden on society [1,2]. In Europe, the estimated prevalence of CRS is 10.9% (6.9 to 27.1%) [3]. CRS comprises heterogeneous diseases with two main phenotypes: with and without nasal polyps (CRSwNP/CRSsNP) [2]. These two phenotypes may be associated with non-steroidal anti-inflammatory drugs (NSAIDs)-exacerbated respiratory disease (N-ERD), asthma, or allergies [2,4]. However, the clinical course of CRS cannot be predicted based on a phenotype alone. The condition can progress or regress, reflected, for instance, by several nasal sinus surgeries indicative of different dynamics of nasal polyps recurrence seen within the phenotype CRSwNP.

CRS can be characterized by a wide range of histopathological findings, specific inflammatory and T-cell patterns, tissue remodeling markers, eicosanoid concentration, and IgE production. Different types of inflammation (type-1, -2, and -3) are associated with CRS to a different degree. The most common is type-2, involving eosinophilic infiltration and production of IL-4, IL-5, and IL-13, but also type-1 (characterized by IFN-γ, TNFα) and type-3 (characterized by IL-17 and IL-22) were described in CRS patients [5,6,7,8]. Both cytokines of type-3 inflammation can be produced either by T cells or by type-3 innate lymphoid cells residing in the mucosal tissues [9], particularly in response to fungal and extracellular bacterial infections; both are associated with chronic inflammation [10]. In addition, IL-17 enhances the epithelial release of antimicrobial peptides, granulopoiesis, neutrophil maturation, and accumulation in peripheral tissues [11], whereas IL-22 induces the expression of Cxcl1, Cxcl2, and Cxcl3 and is involved in the recruitment of neutrophils to the site of skin infection with *Staphylococcus aureus* [12]. In CRS, IL-17 has been associated with the presence of pus in both phenotypes of CRS [13], whereas elevated concentrations of IL-22 are characteristic of CRSwNP [14]. In addition to the above, several other cytokines and their diverse individual and combined contribution to the CRS pathophysiology were recently described in an extensive review [15], adding comprehensive knowledge to the field.

The term “*endotyping*” was introduced by the GA^2^LEN Sinusitis Cohort study of the European FP6-research initiative’s framework to stratify CRS according to histopathological findings [5]. That multicenter case-controlled study primarily aimed to describe CRS endotypes based on immune markers of the nasal sinus tissues obtained from CRS patients. In the cluster analysis, the inflammatory patterns of CRS cases were assigned to CRSsNP and CRSwNP. The IL-5-negative-clusters represented mainly the CRSsNP phenotype, while with an increasing type-2 profile of mild, moderate, and high IL-5 concentrations, the proportion of the CRSwNP phenotype was raised to 100%. The asthma prevalence correlated positively with IL-5 levels and the presence of *Staphylococcus aureus* enterotoxin specific-IgE (SE-IgE) [5]. Therefore, endotyping improved the development of specific and personalized therapeutic strategies. Novel therapies, e.g., with dupilumab (anti-IL-4Rα antibody), omalizumab (anti-IgE antibody), and mepolizumab (anti-IL-5 antibody) target type-2 inflammation in the phenotype CRSwNP [16,17,18]. Biologic treatment is recommended in severe uncontrolled CRSwNP [18,19,20]. However, no biomarker for Th2-targeted biologic therapy has been identified so far.

Chemokines are immune mediators that could contribute to the CRS’s pathology by attracting various effector cells. Chemokines are small peptides that selectively induce the migration of cells bearing the specific receptors and influence their activation status. Chemokines are, in general, involved in specific cell recruitment, inflammation, homeostasis, angiogenesis, wound healing, and leukocyte trafficking [21]. Previous studies revealed that the concentration of the chemokines MIP-1α/CCL3 [22], eotaxin/CCL11 [23], TARC/CCL17 [24], and PARC/CCL18 [25] in tissue samples obtained from CRSwNP patients were higher than in patients with CRSsNP. Also, MCP-3/CCL7 was increased in CRS patients but without significant differences between CRSwNP and CRSsNP [26]. Similarly, the IP-10/CXCL10 concentration was reported to increase in response to viral infection in CRSwNP [27]. There were no differences in ENA-78 levels between CRS patients and healthy subjects [28]. However, none of these studies provided the classification of CRS endotypes in the context of type-1, -2- and -3 inflammation.

In the first primary sub-study of the GA2LEN sinusitis cohort, chemokines were used to control CRS’s endotyping further and set the goals for personalized therapy of CRS. The present study aimed to characterize the expression patterns of various chemokines in the nasal sinus tissues obtained from CRSsNP and CRSwNP patients. The concentration of IL-5 (type-2-related marker) was used as a primary parameter relating the current findings to the past research and allowing the assignment to CRS phenotypes (CRSwNP/CRSsNP) and associated diseases (asthma, N-ERD, and allergies). In addition, we determined the concentrations of CC-chemokines TARC/CCL17, PARC/CCL18, eotaxin/CCL11, MCP-3/CCL7, and MIP-1α/CCL3, and the CXC-chemokines ENA-78/CCL5 and IP-10/CXCL10. Our secondary aim was to examine the association of IL-22 as a marker of type-3 inflammation with the chemokine expression patterns.

## 2. Materials and Methods

### 2.1. Patient Population

The Ethics Committee of the Charité Universitätsmedizin Berlin approved this study (EA2/009/07). All investigations were conducted according to the principles of the Declaration of Helsinki. The patients recruited for the study were diagnosed with CRS based on EPOS criteria [2]. CRS diagnosis was supported by using VAS scales measuring nasal obstruction, secretion in the throat, olfactory symptoms, headache, and facial pressure. In addition, Allergic Rhinitis and its Impact on Asthma (ARIA) guidelines [29] were applied to define allergic subjects. All patients with suspected CRS underwent computed tomography of the head and paranasal sinuses.

Control subjects were asked identical questions concerning symptoms, co-morbidities, current medication, and underwent allergy testing.

All subjects were endoscopically examined directly before surgery, and the control individuals with signs of local inflammation were excluded from the study. All subjects gave their written informed consent. Steroids (oral/nasal) were not administered for four weeks, and leukotriene inhibitors were not used two weeks before surgery. Control and study participants were classified as allergic based on positive skin prick test results or elevated levels of specific IgE to aeroallergens and the presence of allergy symptoms. The presence of allergy was an exclusion criterion for the control subjects. The asthma diagnostics were carried out on all subjects by a pulmonologist. N-ERD diagnosis was based on the medical history of respiratory reactions to NSAIDs.

Nasal biopsies were obtained from 91 CRS patients and control subjects. The CRS group consisted of 66 patients (mean age 42.3 years [min 25, max 65]; 26 females, 40 males). Of them, there were 26 CRSwNP patients (mean age 47.2 years [min 28, max 65] 11 females, 15 males) and 40 patients with CRSsNP (mean age 39.8 years [min 25, max 54]; 15 females, 25 males). The control group consisted of 25 individuals (mean age 31.4 years [min 18; max 60]; 12 females; 13 males).

### 2.2. Patients’ Material

Samples from ethmoidal mucosa of patients with CRSsNP, nasal polyps surgically removed from CRSwNP patients, or mucosal samples from the inferior turbinates in case of controls were collected in saline solution. The tissues were snap-frozen and stored at −80 °C in cryovials. Control tissues were not used for cluster analyses but served as a source for standard values.

### 2.3. Measurement of Inflammatory Markers

According to the study protocol, 100 mg of tissue was diluted in 1 mL of 0.9% NaCl-solution containing protease inhibitor-cocktail (cOmplete™ Protease-Inhibitor-Cocktail, Roche Diagnostics, Mannheim, Germany). Next, the tissue was homogenized (1000 rpm, 5 min, 4 °C) and centrifuged at 1500× *g* for 10 min, 4 °C. The protein concentration was measured using Coomassie-Plus (Bradford) assay kit (ThermoFisher Scientific, Darmstadt, Germany).

The following chemokines were investigated: eotaxin, MIP-1α, MCP-3, ENA-78, TARC, PARC, and IP-10 (Table 1). The chemokine concentration was determined using the commercially available LEGENDplex Multiple Chemokine-Assay (Biolegend, San Diego, CA, USA).

The IL-5- and TNF-α concentrations were measured with the Luminex 100-system (Luminex, Austin, TX, USA). The IL-22, IL-17, and IFN-γ concentrations were determined with ELISA kits from R&D Systems (Minneapolis, MN, USA). The concentration of the tissue eosinophil cationic protein (ECP), total IgE, and IgE directed against a mixture of enterotoxins from *Staphylococcus aureus* (*S. aureus* enterotoxin A, C, and toxic shock syndrome toxin-1) were examined using UniCAP-system (Phadia, Uppsala, Sweden). Myeloperoxidase (MPO) was measured with ELISA (BioCheck, Foster City, CA, USA). The estimated parameters and detection limits are presented in Table 2. Values below the limit of detection were considered negative.

In agreement with the general study protocol [5], the IL-5 levels were retrospectively defined as follows:

- negative IL-5 level when under the concentration of 12.98 pg/mL (assay detection limit)
- low IL-5 level (IL-5 < 100 pg/mL)
- moderate IL-5 level (IL-5 100 to 151 pg/mL)
- high IL-5 level (IL-5 > 151 pg/mL)

### 2.4. Statistical Methods

The following parameters were included in the cluster analysis: IL-22, MPO, eotaxin, IP-10, MCP-3, MIP1α, TARC, ENA-78, PARC, IL-5, total IgE, and tissue ECP. Due to partially strongly right-skewed distributions, all these values were logarithmized. The transformed features were then subjected to a factor analysis based on the Pearson correlation matrix (Extraction Method: Principal Component Analysis, Rotation Method: Varimax with Kaiser Normalization). That resulted in three factors with eigenvalues >1, which explained 66.3% of the total variance. The variable mean values replaced occasional missings within the data. The estimated three-factor scores were then used to perform a hierarchical cluster analysis using the Ward method. Based on the dendrogram, six-well separated clusters could be identified. For the cluster analysis, only the data obtained from the 66 patients and not the controls were used.

The Mann–Whitney U test and the Fisher test were used for the group comparisons between patients and controls, respectively, the exact test variants. The heat map is based on pair comparisons between the six clusters, with 15 pair comparisons for each characteristic. The Mann–Whitney U test was also used in that analysis. These tests have a more descriptive character since the variables involved determined the clusters. An alpha-adjustment for multiple testing was omitted. Overall, the *p*-values of ≤0.05 were set as the significance level. All statistical analyses were performed using IBM-Statistics, Version-24.

## 3. Results

### 3.1. Comparison of CRS and Control Subjects

The control subjects (*n* = 25) were significantly younger (*p* = 0.001; 31.4 yrs vs. 42.3 yrs) than the CRS subjects (*n* = 66). No significant differences regarding gender, smoking, asthma, N-ERD, or allergies were found between CRS cases and controls. Eotaxin, TARC, MPO, total IgE, IL-5, and ECP concentrations were significantly higher in CRS than in the control group (all *p* < 0.005). The other parameters did not significantly differ between the groups (Table 3).

### 3.2. Cluster Analysis Reveals Six Clusters of the Chemokine Expression Pattern

Cluster analysis based solely on chemokine measurements identified six distinct clusters (independent of clinical phenotype). The clusters were well-separated from each other. Means and ratios of chemokines/cytokines (Table 4) and phenotype data were calculated and tabulated as a heat map for cluster characterization (Table 5).

### 3.3. Negative or Low IL-5 Concentration Found in Clusters 1–4

The first cluster was characterized by undetectable IL-5. In Clusters 2–4, IL-5 was detected in low concentration. Additionally, the type-2-associated ECP and total IgE did not differ significantly between Clusters 1–3. ECP and the total IgE were elevated significantly in Cluster 4 (Table 4 and Table 5).

#### Characterization of Clusters

- Cluster-1 (9 CRS cases): No significant differences were detected between the chemokine levels. The concentration of IL-22 (type-3) was significantly higher than in ≥2 clusters (noninflammatory/type-3-based pattern).
- Cluster-2 (11 CRS cases): ENA-78 (neutrophilic parameter), and IP-10 (expressed by type-1) were significantly higher than in ≥2 clusters (neutrophilic, type-1-based inflammatory pattern).
- Cluster-3 (8 CRS cases): The IP-10, MCP-3 (type-1/type-2), and MIP-1α (type-1/type-2) concentrations were significantly higher than in ≥4 clusters. The levels of ENA-78 and type-2-related chemokines TARC and PARC were significantly higher than in the two or more other clusters. IL-22 was significantly higher than in two or more other clusters (mixed neutrophilic type-1- > type-2-, type-3- inflammatory pattern).
- Cluster-4 (13 CRS cases): The concentrations of type-2-related chemokines TARC and PARC were significantly higher than in two or more clusters. ENA-78 was increased significantly compared to two or more other clusters. The concentration of MPO (neutrophilic) was higher than in three or more other clusters, and IL-22 was higher than in two or more other clusters (neutrophilic > type-2 > type-3 inflammatory pattern).

### 3.4. Association of Endotypes with Phenotypes in Clusters 1–4

No nasal polyps were diagnosed in patients belonging to Cluster-2. In Clusters 1, 3, and 4, nasal polyps were found in 11, 13, and 31% of patients, respectively. Asthma, but not N-ERD, was reported in 0 to 23% of patients assigned to Clusters 1 to 4. In Cluster 1, two of nine patients had asthma (22%); in Cluster 2, one of eleven patients had asthma (9%); patients in Cluster 3 had no asthma, and in Cluster 4, three of thirteen patients reported asthma (23%). Asthma predominantly affected patients with CRSwNP. The prevalence of allergic rhinitis in Clusters 1 to 4 ranged between 25 and 62%. The patients in Clusters 1–4 were not previously operated on and underwent their first nasal sinus surgery.

### 3.5. High Concentrations of IL-5: Clusters 5 and 6

In Cluster 5 and 6, the IL-5 and ECP concentrations were significantly higher when compared to the four remaining Clusters.

- Cluster-5 (9 CRS cases): The levels of type-2 related chemokines eotaxin, TARC, and PARC were significantly higher than in four or more clusters. The concentration of MCP-3 was higher than in two other clusters, and MIP-1α was significantly higher than in four or more clusters (type-1, type-2). MPO and IL-22 (type-3) were significantly elevated (three or more other clusters), SAE (type-2) (not included in the cluster analysis) was present (strong Th1/Th2/Th3-pattern).
- Cluster-6 (16 CRS cases): The levels of PARC and eotaxin (four or more other clusters), as well as TARC (two or more other clusters), were significantly elevated. In contrast, no increased concentrations of MCP-3, MIP-1α, ENA-78, or IP-10 were noted. SAE (not included in the cluster analysis) was detected (type-2 inflammatory pattern).

### 3.6. Association of Endotypes with Phenotypes in Clusters 5 and 6

CRS with polyp formation was observed in 78% (Cluster-5) and 81% (Cluster-6) of patients. Asthma was reported by 44% of patients in Cluster-5 and 19% of patients in Cluster-6. All of the asthma-positive patients had CRSwNP. N-ERD was diagnosed in 22% of Cluster-5 patients and 19% of Cluster-6 patients. The prevalence of allergic rhinitis ranged from 11 to 38%, and the mean number of nasal sinus surgeries was 1.7.

## 4. Discussion

Previous research [42] demonstrated that CRS could be subdivided into endotypes based on inflammatory markers. The division of CRS into endotypes helps understand the natural course of the disease and make a treatment choice. Here, we examined the CRS tissues’ chemokine expression to cluster and match the CRS phenotypes CRSwNP/CRSsNP and asthma, N-ERD, and allergic rhinitis. To the best of our knowledge, this is the first study analyzing this panel of chemokines and IL-22 in the context of CRS with primarily endotyping and secondarily phenotyping. The current study allowed associating chemokine expression patterns with the endotypes mentioned above. Additionally, the obtained results demonstrated ex vivo the presence of type-1 inflammation-related biomarkers such as IP-10, neutrophilic biomarker as ENA-78, or TARC, as well as PARC as an early-type-2 inflammation-related biomarker in the CRS nasal tissues. Additionally, IL-22, characteristic of the type-3 immune response, was detected in IL-5-negative/low and IL-5-high clusters. Differences were also found between the clusters with strong type-2 profiles. One cluster was of a pure type-2 profile, whereas IL-22, indicative of type-3, coexisted with neutrophilic inflammation in the other cluster. The identical phenotypes can have different endotypes, and various phenotypes can have the same endotype, highlighting the importance of deep endotyping.

**Figure 1:**
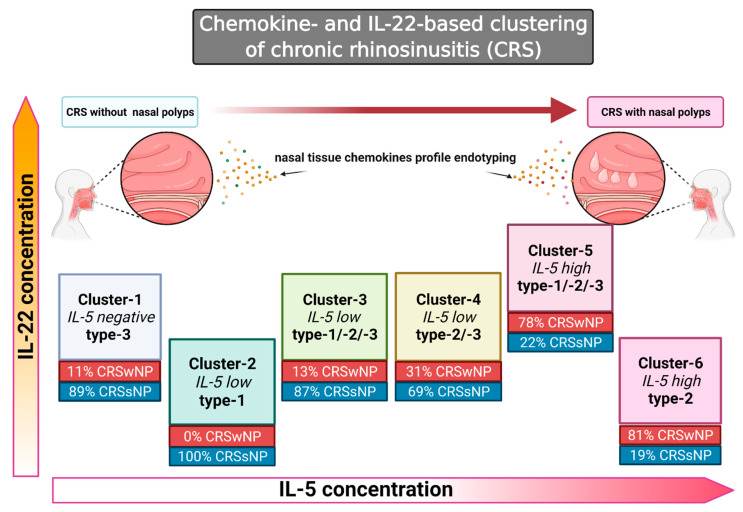
Graphical representation of clusters distribution in relation to the concentration of IL-5 and IL-22 and the occurrence of nasal polyps in the studied cohort of patients with CRS. Created with BioRender.com (accessed on 4 February 2022).

The present study has identified six CRS clusters (Figure 1) based on the tissue chemokine expression pattern in the dataset originating from the multicenter-cooperation GA2LEN “Chronic rhinosinusitis and nasal polyposis cohort study” [5]. Type-2-associated-chemokines and type-1/neutrophilic-chemokines were arbitrarily selected as analytical targets (Table 1).

In Clusters 1–4, the concentration of IL-5 was either low or not detected, consistent with a *noninflammatory* or *low-inflammatory* mixed type-1, -2, and -3 endotypes. Corroborating previous reports [5], noninflammatory chemokines were detected in Cluster-1, in which the IL-22 expression was consistent with type-3 inflammation [43]. A recent report by Vaitkus et al. has associated elevated IL-22 tissue concentration with the presence of nasal polyps in a cohort of patients with CRS [14]. In agreement with that finding, we have observed a positive correlation between very low IL-22 and low IL-5 with the absence of nasal polyps in Cluster-2. However, in Cluster-6, only three of sixteen patients (19%) had no nasal polyps, despite a marginal concentration of IL-22. Nevertheless, Cluster-6 had the highest expression of IL-5, which could have influenced the tissue pathology. Interestingly, the presence of nasal polyps in one of nine patients belonging to the IL-5-negative Cluster-1 demonstrates that the CRSwNP phenotype is not always associated with type-2 endotype, which is of clinical importance when planning biologic therapies.

Cluster-2 was characterized by the presence of elevated concentrations of an epithelial cell-derived neutrophil-activating peptide (ENA-78) and interferon gamma-induced protein 10 (IP-10). ENA-78 attracts and activates neutrophils and can be expressed by the epithelium and activated eosinophils; it is type-3 associated [44] and was suggested to regulate tissue remodeling [45]. The IP-10 expression is induced by type-1-cytokine, IFN-γ. IP-10 does not act on neutrophils but activated T-lymphocytes (type-1 T-helper cells) [41,46,47]. All patients in Cluster-2 (*n* = 11) were free of nasal polyps; thus, all had phenotype CRSsNP.

Cluster-3 was characterized by mixed inflammation (type-1, -2, and -3). The concentration of type-1 and -2 chemokines MCP-3 and MIP-1α was significantly elevated. MCP-3 and MIP-1α predominantly attract type-1/type-2 cells, with MCP-3 attracting monocytes, dendritic cells, lymphocytes, NK-cells, neutrophils, and eosinophils via CCR1, CCR2, and CCR3 [34,35]. In contrast, MIP-1α attracts IFNγ−activated neutrophils as well as a small subpopulation of CCR1-expressing eosinophils [33,48]. In Cluster-3, the TARC and PARC concentrations were significantly higher than in other clusters. Simultaneously, IL-5, ECP, and total IgE concentrations showed no significant differences, suggesting a possibility for using TARC and PARC as early biomarkers of type-2 related inflammation in Cluster-3. In contrast, in Cluster-4, in addition to TARC and PARC, ECP and IgE concentrations were significantly elevated. TARC and PARC are typically produced by dendritic cells and are essential for regulating type-2 immune response and trafficking of memory T-cells, as described in atopic dermatitis [49]. The stimulation of cultured epithelial cells with Th2 cytokines, IL-4 and IL-5, resulted in higher CCL17 expression in nasal polyp epithelial cells when compared to normal ethmoid tissue [50]. In addition, nasal secretions of patients with CRSwNP had higher TARC concentrations than CRSsNP patients [51]. Similar to TARC, the PARC concentration was significantly increased in polyp tissue compared to CRSsNP and controls [25]. Type-2 innate lymphoid cells (ILCs), which were detected in tissues of CRSwNP patients [52], are an essential early source of type-2 cytokines. Murine ILCs2 is derived from pulmonary tissues exposed to allergen-stimulated pulmonary dendritic cells to produce CCL17 [53]. Additionally, IP-10 was found in Cluster 3, indicating type-1 inflammation, while the presence of IL-22, ENA-78, and MPO (also in Clusters-3 and -4), suggested neutrophilic inflammation.

Clusters-5 and -6 had elevated IL-5 concentrations associated with the type-2 inflammatory profile. In addition to TARC and PARC, eotaxin concentration was significantly higher. In CRSwNP patients, eotaxin indicates eosinophilic involvement [32] when compared to controls [23,54]. Jonstam et al. determined that 16 weeks of dupilumab therapy significantly decreases the concentrations of PARC and eotaxin-2 and -3 in nasal secretions and homogenized nasal polyps [55]. Moreover, eotaxin-2 and -3 were reduced in the serum of dupilumab-treated CRSwNP patients versus placebo, demonstrated in a recent phase-3 study [16]. Further, in Cluster-5 (high IL-5), the MCP-3 and MIP-1α [56] concentrations were significantly elevated, pointing to additional type-2 inflammatory effects of these chemokines (35, 36). IL-22 and MPO were increased significantly in Cluster-5, whereas SAE in Clusters-5 and -6. Thus, the patients with strong type-2 inflammation and nasal polyps dichotomize into different endotypes, likely impacting response to biological therapy and possibly prompting future use of different biologics for the treatment of patients belonging to Cluster-5 and Cluster-6.

ENA-78, MPO, and IL-22 were significantly elevated in IL-5-low and -high clusters regarding neutrophilic involvement. Interestingly, Clusters-5 and -6 differed regarding IL-22 expression, and Cluster-6 had a “pure” type-2 profile, whereas Cluster-5 had an additional type-3 component of IL-22 and MPO, indicating a neutrophilic involvement.

Recently, a new endotype in asthma patients was described, where severe neutrophilia is associated with equally severe eosinophilic inflammation and type-3 inflammation. These patients were more affected by asthma severity [57]. IL-22 has proinflammatory and tissue-protective properties depending on the context in which it is expressed [58]. IL-22 inhibits Th_2_–mediated allergic airway inflammation by acting on lung epithelial cells [59], indicative of the protecting role of that cytokine [60]. In contrast, an inflammatory role of IL-22 was described in CRS, indicating that IL-22/IL-22Ra1 axis promotes thymic stromal lymphopoietin (TSLP) production under a type-2 microenvironment in airway epithelial cells, potentially contributing to the development of nasal polyps [60]. In Asian patients with CRS, the expression of IL-22 is associated with IL-17A and TNFα and the absence of nasal polyps [61]. In the present study with Caucasian patients, we have not observed nasal polyps in Cluster-2 with a negligible concentration of IL-22 and type-1 neutrophilic inflammation. Notably, there are known differences between Asian and Caucasian CRS patients regarding inflammatory profiles [6,62], which could account for the seen differences. Two further studies performed by Kim et al. found that the expression of IL-22 in CRSsNP patients could be seen in the presence of all three types of inflammation, whereas in CRSwNP patients, IL-22 correlated with type-2 inflammation [60,63]. In contrast, our study detected IL-22 in IL-5-negative, IL-5-low, and -high clusters. However, we used an entirely different study design and performed cluster analysis in the whole cohort of patients, independent of the phenotypes allocated to the clusters in the later stage. Interestingly, Kim et al. demonstrated that one of the cellular sources of IL-22 in the nasal polyps from the eosinophilic CRS tissues were the mast cells [60], whereas we have not included this type of analysis in our investigation, which is a drawback of this study and needs to be addressed in the future.

Interestingly, the other type-3-related cytokine—IL-17—was either not detected or present in low concentration in the nasal tissues studied here and, therefore, not included in the clustering analysis. That finding agrees with the results of an earlier study, which demonstrated that in the nasal tissues of the CRS patients or control subjects, IL-17 concentrations vary significantly depending on geographic region [64] and that patients originating from Berlin and surroundings have particularly low IL-17. Further studies should clarify the role of type-3 cytokines in CRS.

Corroborating previous results [5], the present study found that the CRSwNP-phenotype correlated positively with the IL-5 concentrations, increasing from 11% in Cluster-1 to 81% in Cluster-6. Moreover, the phenotype CRSwNP with comorbid asthma was mainly associated with a Th2-endotype. The available data confirms the presence of Th2-endotype in patients with CRSwNP and N-ERD. There was no difference in allergic rhinitis prevalence between negative, medium- and highly positive IL-5 clusters. Specific IgE is locally elevated in nasal polyp tissues and is independent of skin-prick test results [42,65]. As described in EPOS2020 [2], this stresses that allergic rhinitis is not necessarily associated with a Th2 endotype of CRSwNP.

In other CRS endotyping work by Delemarre et al., a type-2 inflammation with increased levels of IL-4, IL-5, ECP, IgE, and SAE IgE was observed in 49% of patients with CRSsNP, which is in agreement with the data presented here. Stevens et al. examined the biomarker IFN-γ (type-1), ECP, Charcot-Leyden crystal galectin (type-2), and IL-17A (type-3) and found the type-3 markers in CRSsNP and CRSwNP [13]. Klingler et al. performed an endotyping of CRSsNP and found a rate of detectability of IP-10 in 100% of the CRSsNP samples, similar to our data demonstrating IP-10 (type-1 biomarker) in the “pure” type-1 profile. Klingler described the detection rates of the proteins ECP, IL-13, and CCL26 in nasal lavage fluid of patients in CRSsNP to be 100%, 14.0%, and 75.6%, respectively [66].

The limitations of the present study include the relatively small number of patients, and future multicenter studies should address this pitfall. The other shortcoming of our study is that the N-ERD diagnosis was only based on the clinical history, and controlled provocations with NSAIDs performed in the clinical settings could improve or confirm the diagnosis. Another drawback of the present study is that the tissues from the control subjects (inferior turbinate) could also possibly be a site of immunological activities at the time of resection, as they are continuously exposed to the environment. Finally, our research design has not exhausted all analytical possibilities and left open the data exploration primarily based on clinical symptoms (e.g., presence of polyps or asthma). Further, an exploration of patients’ individual symptom profiles and their relation to cytokine and chemokine expression may complete our understanding.

## 5. Conclusions

In conclusion, the CRS endotype clustering based on a chemokine expression pattern has confirmed and extended previous findings [5]. We identified detailed CRS endotypes characterized by a wide diversity of inflammatory markers. Increased TARC, PARC, and eotaxin levels associated with type-2 biomarkers in CRSwNP. TARC and PARC could serve as early markers in patients without upregulation of IL-5 and IgE, improving the decision-making process in therapy with biologics. Using additional neutrophilic biomarkers such as ENA-78 contributes to further CRS endotype characterization. IL-22 was detected in all IL-5 groups (IL-5 negative, low and high), possibly indicating a regulatory role in the noninflammatory endotype and an inflammatory role in the high IL-5 endotype.

Furthermore, patients with severe CRSwNP and type-2 profiles differed concerning neutrophil type-3 inflammatory markers, indicating the presence of diverse endotypes within one phenotype. Therapeutic options are likely required to address intense neutrophilic inflammation in severe cases of CRSwNP. This study’s benefits include providing evidence for new biomarkers useful for personalized diagnosis and monitoring and may open the path for relevant sub-endotypes within the CRSwNP patients in the future. Our observations deepen the knowledge about CRS endotypes based on cardinal cytokine expression and should be further expanded to increase the general knowledge, augment the diagnostic and monitoring possibilities, and tune up the therapeutic approach. The recent development of biologics therapy for upper respiratory tract disorders fully justifies the individual, personalized diagnostic approach to warrant a successful treatment and monitoring of CRS.

## Figures and Tables

**Table 1:** Source and function of analyzed chemokines.

Chemokine	Motif	Receptor	Origin	Main Immune Function
Eotaxin-1/CCL11	CC	CCR3	secreted by epithelial, mesenchymal, endothelial cells, and eosinophils [30]	attracts eosinophils [31] and basophils, selective on CCR3 receptor [32]
MIP1α/CCL3 (macrophage inflammatory protein-1α)	CC	CCR1, CCR5	secreted by T- and B-lymphocytes, neutrophils, dendritic cells, mast cells, and NK cells [33]	attracts IFNγ-activated neutrophils and a small subpopulation of CCR1 expressing eosinophils [33]
MCP-3/CCL7 (monocyte chemotactic protein-3)	CC	CCR1, CCR2, CCR3	secreted by mononuclear cells [34]	attracts monocytes, dendritic cells, lymphocytes, NK cells, eosinophils, basophils, and neutrophils [35]
ENA-78/CXCL5 (epithelial-derived neutrophil-activating peptide 78)	CXC	CXCR2	secreted by monocytes, endothelial cells, mast cells, keratinocytes, fibroblasts [36]	attracts neutrophils [36]
TARC/CCL17 (thymus- and activation-regulated chemokine)	CC	CCR4	secreted by monocytes, dendritic and epithelial cells [24]	attracts T lymphocytes (Th2)
PARC/CCL18 (pulmonary and activation- regulated chemokine) [37]	CC	CCR8	secreted by dendritic cells, monocytes, macrophages, mast cells, eosinophils, and neutrophils [38]	attracts naive CD4+ and CD8+ T cells, B cells, and immature dendritic cells [37,39]
IP-10/CXCL10 (IFNγ-inducible protein 10)	CXC	CXCR3	secreted by monocytes, T-lymphocytes, fibroblasts, keratinocytes [40] in response to the Th1-cytokine IFNγ	attracts activated Th1-lymphocytes [41]

**Table 2:** Markers used for the component and cluster analysis, the cutoff values for markers analyzed as categorical.

Marker	Cutoff Values *	Interpretation of Increased Concentrations
Eotaxin/CCL11	NA	Type-2 immunity
MCP-3/CCL7	NA	Associated with type-1 and type-2 immunity
MIP-1 α/CCL3	NA	Associated with type-1 and type-2 immunity
ENA-78/CXCL5	NA	Neutrophilic inflammation
TARC/CCL17	NA	Type-2 immunity
PARC/CCL18	NA	Type-2 immunity
IP-10/CXCL10	NA	Type-1 immunity
ECP	NA	Type-2 immunity
IgE	NA	Type-2 immunity
SE-IgE	3.85 kU_A_/L *	Marker of superantigen effect on local mucosa
IL-5	12.98 pg/mL *	Type-2 immunity
MPO	NA	Neutrophilic activity
IL-22	NA	Type-3 immunity
TNF-α	38.94 pg/mL *	Type-1 immunity
IL-17	25.06 pg/mL *	Type-3 immunity
IFN-ɣ	85.8 pg/mL *	Type-1 immunity

* Cutoff values according to Tomassen et al., 2016 [5].

**Table 3:** Descriptive statistics of CRS patients and control subjects.

		Patients	Controls	Significance
		MV Min–Max (SD)	MV Min–Max (SD)	
	Number	66	25	
Clinic	Age (mean)	42.3 25–65 (10.0)	31.4 18–60 (12.1)	*p* < 0.001 ^1^
	Gender male %	62.1	52.0	*p* = 0.475 ^2^
	Ever smoked %	69.7	48.0	*p* = 0.086 ^2^
	Smoker current %	39.4	36.0	*p* = 0.814 ^2^
	Allergy %	33.3	16.0	*p* = 0.124 ^2^
	Asthma %	19.7	8.0	*p* = 0.222 ^2^
	N-ERD %	7.6	0.0	*p* = 0.317
Laboratory Findings	Eotaxin/CCL11 pg/mL	37.7 2.5–677.7 (85.9)	11.7 1.8–52.5 (11.6)	*p* = 0.001 ^1^
	MCP-3/CCL7 pg/mL	3.3 0.4–14.3 (2.5)	5.7 0.8–47.5 (12.1)	*p* = 0.147 ^1^
	MIP-1α/CCL3 pg/mL	3.8 0.9–19.3 (3.1)	5.9 1.4–45.8 (9.5)	*p* = 0.545 ^1^
	ENA-78/CXCL5 pg/mL	43.0 3.7–215.6 (49.6)	113.2 5.9–1263.5 (262.4)	*p* = 0.140 ^1^
	TARC/CCL17 pg/mL	8.3 1.4–40.9 (8.0)	5.9 0.8–54.9 (12.8)	*p* < 0.001^1^
	PARC/CCL18 pg/mL	409.4 10.8-3246.5 (626.7)	119.0 40.2-235.6 (50.8)	*p* = 0.016 ^1^
	IP-10/CXCL10 pg/mL	330.5 15.2-4510.2 (620.9)	274.3 25.0-1208.4 (257.4)	*p* = 0.240 ^1^
	Total IgE kU/L	262.2 0.0–3641.0 (604.3)	30.0 1.7–293.7 (60.6)	*p* = 0.001^1^
	ECP pg/mL	4971.0 11.0–32,725.0 (7499.4)	194.4 11.0–1254.0 (313.6)	*p* < 0.001 ^1^
	IL-5 pg/mL	136.5 6.5–963.7 (215.6)	6.5 6.5–6.5 (0)	*p* < 0.001 ^1^
	SAE kU/L	0.8 0.0–8.7 (2.1)	0.0 0.0–0.0 (0.0)	*p* = 0.095 ^1^
	IL-22 pg/mL	537.1 140.3–1680.5 (294.4	494.6 254.7–1473.9 (256.2)	*p* = 0.577 ^1^
	MPO ng/mL	4198.1 166.2–56,996.5 (7560.1)	1160.4 158.1–4683.1 (1100.7)	*p* < 0.001 ^1^
	TNF-α pg/mL (>Threshold %)	3.0	0.0	*p* = 1.000 ^2^
	IL-17 pg/mL (>Threshold %)	4.5	4.0	*p* = 1.000 ^2^
	IFN-γ pg/mL (>Threshold %)	9.7	4.2	*p* = 0.668 ^2^

^1^ Exact Mann–Whitney U Test (two-tailed); ^2^ Exact Fisher Test (two-tailed).

**Table 4:** Endotype profiling.

	Variables Used in Cluster Analysis												Variables Not Used in Cluster Analysis					
	ENA_78/CXCL5	MPO	IP-10/CXCL10	MIP1a/CCL3	MCP-3/CCL7	TARC/CCL17	PARC/CCL18	Eotaxin/CCL11	ECP	IL-5	Total-IgE	IL-22	SAE	SAE Ratio	Ratio IL5	TNF-α-Categorial	IL-17-Categorial	IFN-γ-Categorial
Cluster (Number of Cases)	Mean	Mean	Mean	Mean	Mean	Mean	Mean	Mean	Mean	Mean	Mean	Mean	Mean	Ratio Positive	Ratio Positive	Ratio Positive	Ratio Positive	Ratio Positive
1 (*n* = 9)	32.91	2866.26	332.78	1.26	1.36	3.33	80.58	6.57	686.46	6.49	42.74	693.74	0.00	0.00	0.00	0.00	0.00	0.29
2 (*n* = 11)	53.37	1982.33	346.33	2.91	3.02	3.16	127.10	9.59	373.86	30.69	17.19	372.86	0.00	0.00	0.18	0.00	0.09	0.00
3 (*n* = 8)	82.27	2426.66	1110.10	8.49	6.60	6.32	168.23	18.47	1294.99	17.74	66.89	530.65	0.00	0.00	0.25	0.00	0.00	0.00
4 (*n* = 13)	45.04	4034.73	264.41	2.61	2.65	6.77	155.50	21.37	2673.59	83.46	142.84	640.02	0.00	0.00	0.54	0.08	0.15	0.08
5 (*n* = 9)	59.712	13031.6	142.68	6.34	4.72	21.44	1072.15	114.79	12793.00	204.76	710.55	981.71	2.34	0.33	0.89	0.11	0.00	0.13
6 (*n* = 16)	10.73	2795.16	87.72	3.10	2.65	9.57	742.61	54.09	9846.44	346.29	524.4	312.85	1.46	0.27	1.00	0.00	0.00	0.13

Red fields indicate concentration significantly higher than four or more other clusters. Blue fields indicate concentration significantly higher than three other clusters. Orange fields indicate concentration significantly higher than two other clusters.

**Table 5:** Association of the endotypes with the clinical phenotypes. Simplified schema of the clusters and their characteristic chemokines and the proportion of CRSwNP and CRSsNP, asthma, and N-ERD. One cluster with negative IL-5, 3 Clusters with low IL-5, and 3 Clusters with high IL-5 values are given.

Cluster	Number of Cases	IL-5	IL-5 Positive Ratio %	IL-5	ECP	Total IgE	PARC (CCL18)	TARC (CCL17)	Eotaxin (CCL11)	MCP3 (CCL7)	MIP1α (CCL3)	ENA-78 (CCL5)	MPO	IP-10 (CXCL10)	IL-22	NP	Average Number of Surgeries	Asthma	Allergic Rhinitis	N-ERD
1	9	negative	0.0%												>2	1/9 (11%)	1	2/9 (22%)	3/9 (33%)	0/9 (0%)
2	11	low	18.2%									>2		> 2		0/11 (0%)	1	1/11 (9%)	3/11 (27%)	0/11 (0%)
3	8		25.0%				>2	>2		>4	>4	>2		>4	>2	1/8 (13%)	1	0/8 (0%)	2/8 (25%)	0/8 (0%)
4	13		53.8%		>2	>2	>2	>2				>2	>3		>2	4/13 (31%)	1	3/13 (23%)	8/13 (62%)	0/13 (0%)
5	9	high	8.9%	>4	>4	>4	>4	>4	>4	>2	>4		>3		>3	7/9 (78%)	1.7	4/9 (44%)	1/9 (11%)	2/9 (22%)
6	16		100.0%	>4	>4	>3	>4	>2	>4							13/16 (81%)	1.7	3/16 (19%)	6/16 (38%)	3/16 (19%)

Simplified presentation of the clusters and their characteristic chemokines with the proportion of CRSwNP and CRSsNP, asthma, and N-ERD. There was one cluster with negative IL-5, three clusters with low IL-5, and two clusters with high IL-5 concentrations. The severity of significant increases between CRS cases are as follows: >2: concentration significantly higher than two other clusters; >3: concentration significantly higher than three other clusters; >4: concentration significantly higher than four or more other clusters.

## Data Availability

Data is available on request.
